# Cytoplasmic Accumulation of Heterogeneous Nuclear Ribonucleoprotein K Strongly Promotes Tumor Invasion in Renal Cell Carcinoma Cells

**DOI:** 10.1371/journal.pone.0145769

**Published:** 2015-12-29

**Authors:** Taiyo Otoshi, Tomoaki Tanaka, Kazuya Morimoto, Tatsuya Nakatani

**Affiliations:** Department of Urology, Osaka City University Graduate School of Medicine, Osaka, Japan; Peking University Cancer Hospital & Institute, CHINA

## Abstract

Heterogeneous nuclear ribonucleoprotein (hnRNP) K is a part of the ribonucleoprotein complex which regulates diverse biological events. While overexpression of hnRNP K has been shown to be related to tumorigenesis in several cancers, both the expression patterns and biological mechanisms of hnRNP K in renal cell carcinoma (RCC) cells remain unclear. In this study, we showed that hnRNP K protein was strongly expressed in selected RCC cell lines (ACHN, A498, Caki-1, 786–0), and knock-down of hnRNP K expression by siRNA induced cell growth inhibition and apoptosis. Based on immunohistochemical (IHC) analysis of hnRNP K expression in human clear cell RCC specimens, we demonstrated that there was a significant positive correlation between hnRNP K staining score and tumor aggressiveness (e.g., Fuhrman grade, metastasis). Particularly, the rate of cytoplasmic localization of hnRNP K in primary RCC with distant metastasis was significantly higher than that in RCC without metastasis. Additionally, our results indicated that the cytoplasmic distribution of hnRNP K induced by TGF-β stimulus mainly contributed to TGF-β-triggered tumor cell invasion in RCC cells. Dominant cytoplasmic expression of ectopic hnRNP K markedly suppressed the inhibition of invasion by knock-down of endogenous hnRNP K. The expression level of matrix metalloproteinase protein-2 was decreased by endogenous hnRNP K knock-down, and restored by ectopic hnRNP K. Therefore, hnRNP K may be a key molecule involved in cell motility in RCC cells, and molecular mechanism associated with the subcellular localization of hnRNP K may be a novel target in the treatment of metastatic RCC.

## Introduction

Renal cell carcinoma (RCC) comprises a major portion of malignant neoplasms of the kidney [1]. It is the seventh most common cancer in men and the ninth in women [2]. Approximately 30% of patients with RCC exhibit metastasis, and the 5-year survival of these patients with metastatic RCC has been reported to be less than 10% [3,4].

Several alternative treatments have recently been developed for metastatic RCC. Vascular endothelial growth factor (VEGF) is a potent pro-angiogenic protein, which is responsible for increased vasculature and tumor growth in RCC. Basically, a mutation in the von Hippel-Lindau (VHL) tumor suppressor gene induces overexpression of VEGF via accumulation of hypoxia-inducible factor (HIF)-1 in RCC, particularly clear cell carcinoma [5,6]. Several agents inhibiting the VEGF signaling cascade, such as sorafenib, sunitinib, axitinib, pazopanib and bevacizumab, have been found to exert significant anti-tumor effects and provide meaningful clinical benefit [7,8,9,10,11]. Furthermore, temsirolimus and everolimus, inhibitors of the mammalian target of rapamycin (mTOR) which block the phosphoinositide 3-kinase (PI3K)/AKT signaling pathway involved in diverse cellular functions including cell proliferation, survival and angiogenesis, have been found to be effective agents against advanced RCC in clinical settings [12,13]. While these molecular targeted therapies against the VEGF or mTOR signaling pathway have revolutionized the treatment of advanced RCC, no curative therapy has yet been established because RCC cells acquire resistance to these targeted treatments over a few years [14,15].

The heterogeneous nuclear ribonucleoprotein (hnRNP) K, a component of the hnRNP complex, is a highly conserved RNA- and DNA-binding protein. It is composed of 464 amino-acid residues with a calculated molecular mass of 48–51 kDa. Structurally, it contains three consecutive K homologue (KH) domains that are responsible for the binding of RNA or single-stranded DNA, a nuclear localization signal (NLS) serving upon its transport from the cytoplasm to the nucleus, and a nuclear shuttling domain (KNS) that promotes bi-directional nucleo-cytoplasmic shuttling via the nuclear pore complex [16,17,18]. Biologically, it interacts with diverse molecules involved in gene expression and signaling pathways in biological events such as chromatin remodeling, RNA processing, RNA splicing, RNA stability, translation and post-translational modification [19]. Expression of several oncogenes (e.g., c-Src, c-myc, eIF4E) has been shown to be regulated by hnRNP K [20,21,22]. On the other hand, hnRNP K has been identified as a HDM2-target molecule and mediates transcriptional responses to DNA damage in cooperation with p53 protein [23,24]. Moreover, expression of hnRNP K has been found to be upregulated in many cancers including lung, oral, breast, colorectal, hepatic, pancreatic, and prostate cancer and melanoma [25,26,27,28,29,30,31]. In particular, increased cytoplasmic distribution of hnRNP K has been shown to be positively related to tumor aggressiveness and poor clinical outcomes in some cancers [29,32,33]. Thus, hnRNP K is a crucial player in tumor progression and malignant potency. However, there is no report on the biological role of hnRNP K in human RCC.

In this study, we examined the altered expression of hnRNP K protein in human RCC cell lines. We next investigated the effect of endogenous hnRNP K knock-down on these RCC cells. Immunohistochemical analysis of RCC specimens showed a positive correlation of expression level with cancer grade and metastasis. There was also increased cytoplasmic hnRNP K expression in primary RCC with distant metastasis. Furthermore, we tested the effect of hnRNP K knock-down on TGF-β-induced cell invasion through the regulation of cellular localization of hnRNP K expression in RCC cells. Finally, we examined whether exogenous mutant hnRNP K protein, which has the ability of cytoplasmic accumulation, directly controls cell invasion.

## Materials and Methods

### Antibodies

Mouse monoclonal anti-β-actin and anti-histone H1 antibodies, and rabbit polyclonal anti-calpain antibody were obtained from Abcam (Cambridge, UK). Rabbit polyclonal anti-hnRNP K (R332) and anti-matrix metalloproteinase protein (MMP)-2 antibodies were from Cell Signaling Technology (Beverly, MA, USA). The hnRNP K (R332) antibodies are produced by immunizing rabbits with a synthetic peptides corresponding to residues surrounding Arginine 332 of human hnRNP K protein, and purified with protein A and peptide affinity chromatography. Thus, hnRNP K (R332) antibody can detect specifically endogenous level of total hnRNP K protein. Mouse monoclonal anti-FLAG antibody was from SIGMA-ALDRICH (St. Louis, MO, USA).

### hnRNP K silencing using siRNAs

hnRNP K short interfering RNAs (si-hnRNP K) (s6738 and s6739) and negative control siRNAs were purchased from Ambion by Life Technologies (Carlsbad, CA, USA). Each siRNA was transfected into sub-confluent cells using Lipofectamine RNAiMAX (Invitrogen by Life Technologies) according to the manufacturer’s instructions. At 72 h after transfection, the transfected cells were harvested, followed by western blotting or invasion assay.

### Cell culture and reagents

Human RCC cell lines, ACHN (CRL-1611), Caki-1(HTB-46), A498 (HTB-44) and 786–0 (CRL-1932) were obtained from the American Type Culture Collection (Manassas, VA, USA). The Caki-1 and A498 and ACHN cell lines were maintained in Dulbecco’s Modified Eagle’s Medium (Sigma, St. Louis, MO, USA), and 786–0 cells were maintained in RPMI-1640 (Sigma) supplemented with 10% fetal bovine serum (HyClone, Logan, UT, USA), 100 U/ml penicillin and 100 μg/ml streptomycin (Gibco, New York, NY, USA) at 37°C in a humidified atmosphere containing 5% CO2. The human renal proximal tubule epithelial cell line, RPTEC (Lonza, Basel, Switzerland), was maintained in REGM Bulletkit (Lonza). TGF-β was purchased from Cell Signaling Technology (Beverly, MA, USA) and dissolved in citric acid. The concentration of stock solution was 50 μg/ml, and the final concentration was 10 ng/ml for all experiments.

### Clinical specimens and immunohistochemical staining

Sixty five clear cell type RCC samples and seven normal renal specimens from kidneys diagnosed with angiomyolipoma (AML), which were collected by nephrectomy between 2010 and 2014 at Osaka City University, were used for immunohistochemical (IHC) examination.

The study was approved by the ethics committee of Osaka City University Hospital. Written informed consent was obtained from all patients participating in this study.

After de-paraffinization, specimens were blocked with 10% goat serum for 60 min, and incubated with primary anti hnRNP K antibody (1:100, Cell Signaling Technology, Danvers, USA) at 4°C overnight and then incubated with the secondary antibody containing avidin-biotin-peroxidase complex. DAB solution (3,3’-diaminobenzidine) was used for 2 min to visualize a brown color. The sections were counterstained with hematoxylin (Wako, Osaka, Japan) for 25 sec.

The expression of hnRNP K was evaluated by the staining intensity and the percentage of positive cells. The staining intensity was differentiated into three grades: 1 (weak), 2 (moderate), and 3 (strong). The percentage was also scored into four categories: 1 (<20%), 2 (20–49%), 3 (50–79%), and 4(>80%). The sum of the intensity grade and the percentage score was determined as the final score. The immunostained specimens were independently evaluated by two observers (T.O. and T.T.). For most of the results, there was no difference between the two observers. In the case of disagreement, the final score was determined as the collective opinion.

### Western blotting

Cells were harvested and whole-cell lysates were prepared using PRO-PREP protein extraction solution (iNtRON Biotechnology, Gyeonggi-do, Korea). To separate the nuclear and cytoplasmic fractions from the total cell lysate, a nucleus/cytosol fractionation kit (BioVision, Milpitas, CA, USA) was used in accordance with the manufacturer’s instructions. Protein concentration of samples was determined by the bicinchoninic acid protein assay (BioRad, Hercules, CA, USA). Protein samples were treated at 55°C for 10min in 2% SDS solution containing 5% β-mercaptoethanol, separated in 10% SDS-polyacrylamide gels, and transferred onto nitrocellulose membranes. Membranes were blocked for 1 h at room temperature with Tris-buffered saline (TBS) containing 0.05% Tween 20 and 5% non-fat dried milk, and probed overnight at 4°C with primary antibodies. Immunoblots were washed with TBS containing 0.05% Tween 20 and 1% non-fat milk, and incubated with secondary antibodies conjugated with horseradish peroxidase against mouse IgG or rabbit IgG (Santa Cruz Biotechnology, Santa Cruz, CA, USA) for 1 h at room temperature. Immunoreactive proteins were visualized using the ECL detection system (Pierce, Rockford, IL, USA). Each western blot analysis was performed in triplicate.

### Cell proliferation assay

Cells (3.0×10^4^ cells per well) were placed into 500 μl of medium in 24-well plates (Corning Inc., New York, NY, USA). After culturing for 24, 48, 72, 96 or 120 h, the supernatant was removed, and cell-growth inhibition was analyzed using the Premix WST-1 cell proliferation assay (TAKARA Bio, Otsu, Japan) according to the manufacturer’s instructions. Absorbance was measured at 450 nm using a microplate reader (Perkin Elmer Inc., Waltam, MA, USA). All assays were carried out in triplicate.

### Apoptosis analysis

Cells were transfected with 9 μl of siRNA as negative control or hnRNP K siRNA using 45 μl lipofectamin RNAiMAX (Invitrogen Life Technologies) according to the manufacturer’s protocol. The transfectants were stained at 24 h after transfection using an APO-DIRECT kit (BD Biosciences, San Diego, CA, USA). Cells were washed in PBS and resuspended in 1% (w/v) paraformaldehyde in PBS. After incubation on ice, aliquots of cells were centrifuged and resuspended in 70% (v/v) ice-cold ethanol. The cell suspensions were incubated in staining solution containing fluorescein isothiocyanate-dUTP, terminal deoxynucleotidyl transferase enzyme and reaction buffer for 1h at 37°C. After washing with rinse buffer, the treated cells were finally stained with propidium iodide. Subsequently, all samples were analyzed with a FACSCalibur flow cytometer with CellQuest software (BD Biosciences, Mountain View, CA, USA).

### Plasmid constructs and site-directed mutagenesis

The plasmid vectors, pCMV6-Entry hnRNP K and pCMV6-Entry empty vector, were purchased from Origene (catalogue no RC201843 and PS100001, respectively). Based on the information of the nuclear localizing signal sequence [34], the cDNA of hnRNP K mutant was generated in hnRNP K at Lys-21 and Arg-22 by PCR-based site-directed mutagenesis using appropriate primers, followed by ligation with the vector, pcMV6-Entry-hnRNP K as described previously [35,36]. Primers used to mutate Lys-21 to Ala and Arg-22 to Ala were as follows: 5'- aat ggt gaa ttt ggt gca gcc cct gca gaa gat atg-3' and 5'- cat atc ttc tgc agg ggc tgc acc aaa ttc acc att -3'. Primers used to generate siRNA-resistant hnRNP K were 5'- caa atc cgt cat gag tcg ggc gcc tca ata aag ata gac gag cct tta gaa gga tcg gat c-3' and 5'- gat ccg atc ctt cta aag gct cgt cta tct tta ttg agg cgc ccg act cat gac gga ttt g-3'. The sequences of the inserts were confirmed by DNA sequencing analysis. To express FLAG-tagged hnRNP K, hnRNP K mutant, si-hnRNP K-resistant FLAG-hnRNP K and si-hnRNP K-resistant FLAG-mutant hnRNP K, the plasmids described above were transfected into RCC cells using Lipofectamine 2000 (Invitrogen Life Technologies) according to the manufacturer’s instructions.

### Transwell invasion assay

The invasion assay employed Corning Matrigel invasion chambers (8 μM pore size; BD Biosciences). Cells (5.0×10^4^ cells/500 μl serum-starved medium) were added to the upper chamber. Complete medium was added to the bottom wells of the chambers. At 24 h after the start of this treatment, the supernatant was removed from the upper chamber, and the upper face of the filters was wiped using cotton swabs. The cells that had infiltrated were fixed with 5% glutaraldehyde solution and stained with Giemsa stain solution. Images of four different ×100 fields were captured from each membrane and the number of invading cells was counted.

### Statistical analysis

Statistical analysis was performed using JMP 10.0 software (SAS Institute, Cary, NC, USA). Data were expressed as mean ± S.D. Student's *t*-test was used to calculate the statistical significance of the experimental results of invasion assays and proliferation assays. Mann Whitney *U*-test and Kruskal-Wallis one-way analysis were used for analysis of scores of immunohistochemical staining. The significance level was set as *P* < 0.05.

## Results

### Down-regulation of hnRNP K expression suppresses cell proliferation in RCC cells

**Fig 1:**
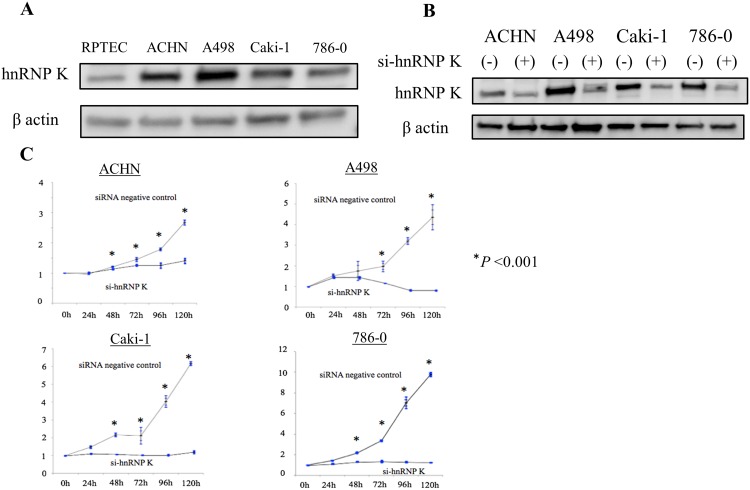
Inhibition of proliferation in RCC cells by hnRNP K knocking down. Expression of heterogeneous nuclear ribonucleoprotein (hnRNP) K in both normal renal proximal tubule cell line (RPTEC) and renal cell carcinoma (RCC) cell lines, and growth inhibition of four RCC cell lines after knock-down of endogenous hnRNP K. As described in the Materials and Methods section, the number of viable RCC cells after treatment with control siRNA or siRNA against hnRNP K (si-hnRNP K) for 0, 24, 48, 72, 96 or 120 h was determined by water-soluble tetrazolium salt (WST-1) assay. A. Total cell lysates were prepared from each cell line and analyzed by western blotting using anti-hnRNP K antibody to detect hnRNP K protein (upper panel). To elucidate equal loaded amounts of total cell lysates, western blotting using anti-β-actin antibody was also performed (lower panel); B. Each RCC cell line transfected with negative control siRNA or si-hnRNP K was harvested 72 h after transfection and total cell lysates were analyzed by western blotting using anti-hnRNP K antibody to evaluate the efficacy of si-hnRNP K; C. Values measured at 0 h with control siRNA or si-hnRNP K were determined as 1. Three individual experiments were performed. Results were presented as mean ± S.D. *P<0.05 compared with control (Student’s *t*-test, n = 3).

Protein expression of hnRNP K in both a normal human renal cell line (RPTEC) isolated from renal proximal tubule and four RCC cell lines (ACTH, A498, Caki-1, 786–0) was determined by Western blot analysis using anti-hnRNP K antibody. As shown in Fig 1A, the expression levels of hnRNP K in these RCC cell lines were very high as compared with that in normal renal cells. Next, we examined the effect of hnRNP K knock-down on cell growth in RCC cells using siRNA against hnRNP K (si-hnRNP K). First, we checked the knock-down effect of two idependent si-hnRNP K (s6738 and s6739) in A498 cells. As the knocking down efficiency of s6739 was superior to that of s6738 (S1 Fig), we used this si-hnRNP K (s6739) in the latter experiments. In the four RCC cell lines transfected with si-hnRNP K, the amount of hnRNP K protein was reduced to less than 20% of that in control RCC cells (Fig 1B). In the si-hnRNP K-mediated cell-growth assay, proliferation of ACHN, Caki-1 and 786–0 cells was significantly suppressed compared with that of control after culture for 48 h. On the other hand, growth of A498 cells was significantly reduced compared with that of control cells after culture for 72 h (Fig 1C).

### Down-regulation of hnRNP K expression induces apoptosis in A498 and Caki-1 RCC cells

**Fig 2:**
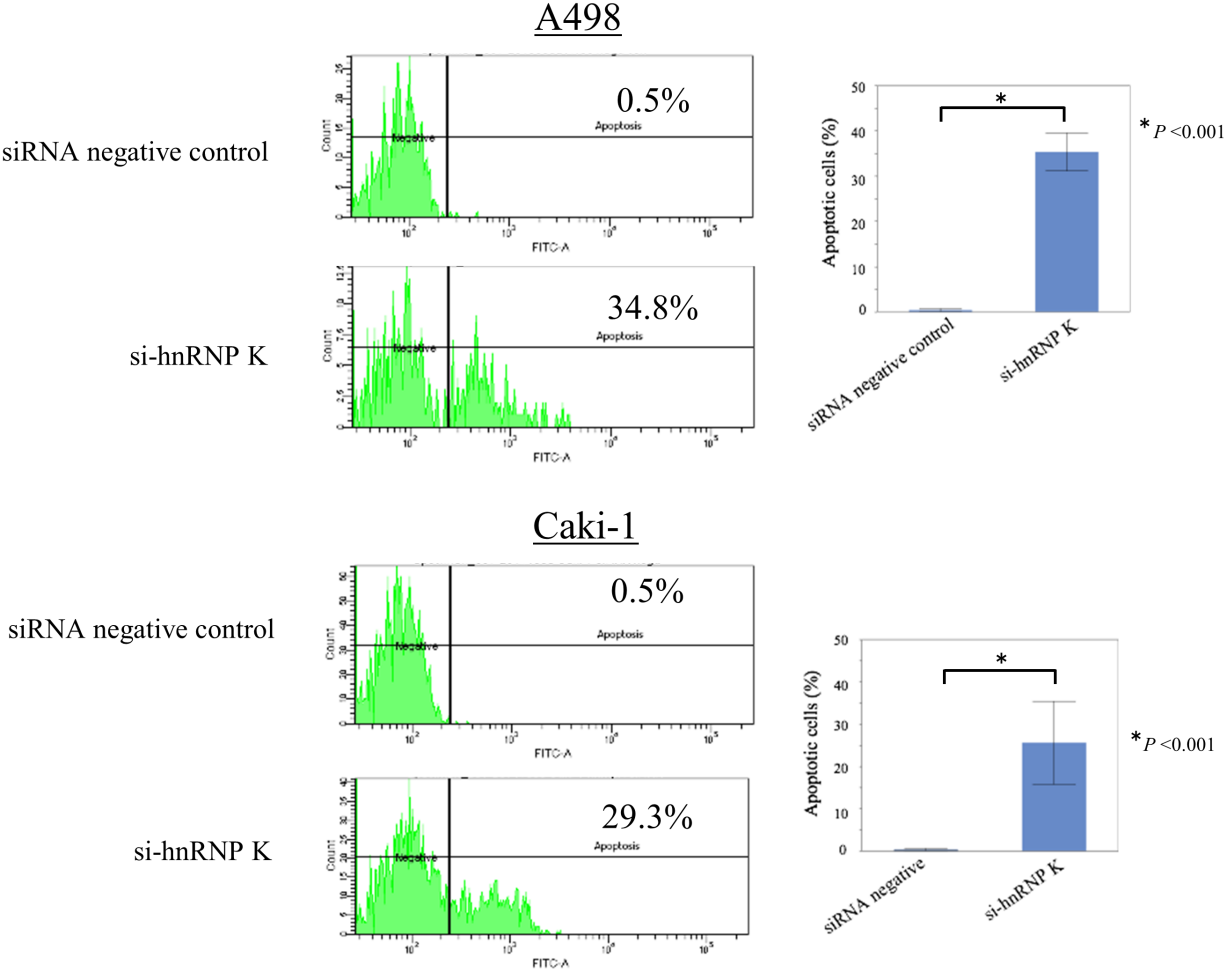
Induction of apoptosis by hnRNP K knocking down in RCC cells. Measurement of apoptotic cells in A498 (upper panel) and Caki-1 (lower panel) cell lines transfected with control siRNA or si-hnRNP K using flow cytometry. Three individual experiments were performed, and the percentage of apoptotic cells in these cell lines is presented as means ± s.d. *P<0.05 compared with control (Student’s *t*-test, n = 3).

We investigated whether hnRNP K knock-down induces apoptosis in RCC cells, using flow cytometry 72 h after transfection with si-hnRNP K or control siRNA. Knock-down of endogenous hnRNP K markedly increased the rate of apoptotic cells compared with control; 34.8% (p<0.001) and 29.3% (p<0.001) in A498 and Caki-1 cells, respectively (Fig 2).

### Expression and subcellular distribution of hnRNP K protein positively correlate with pathological grade and distant metastasis in RCC specimens

**Fig 3:**
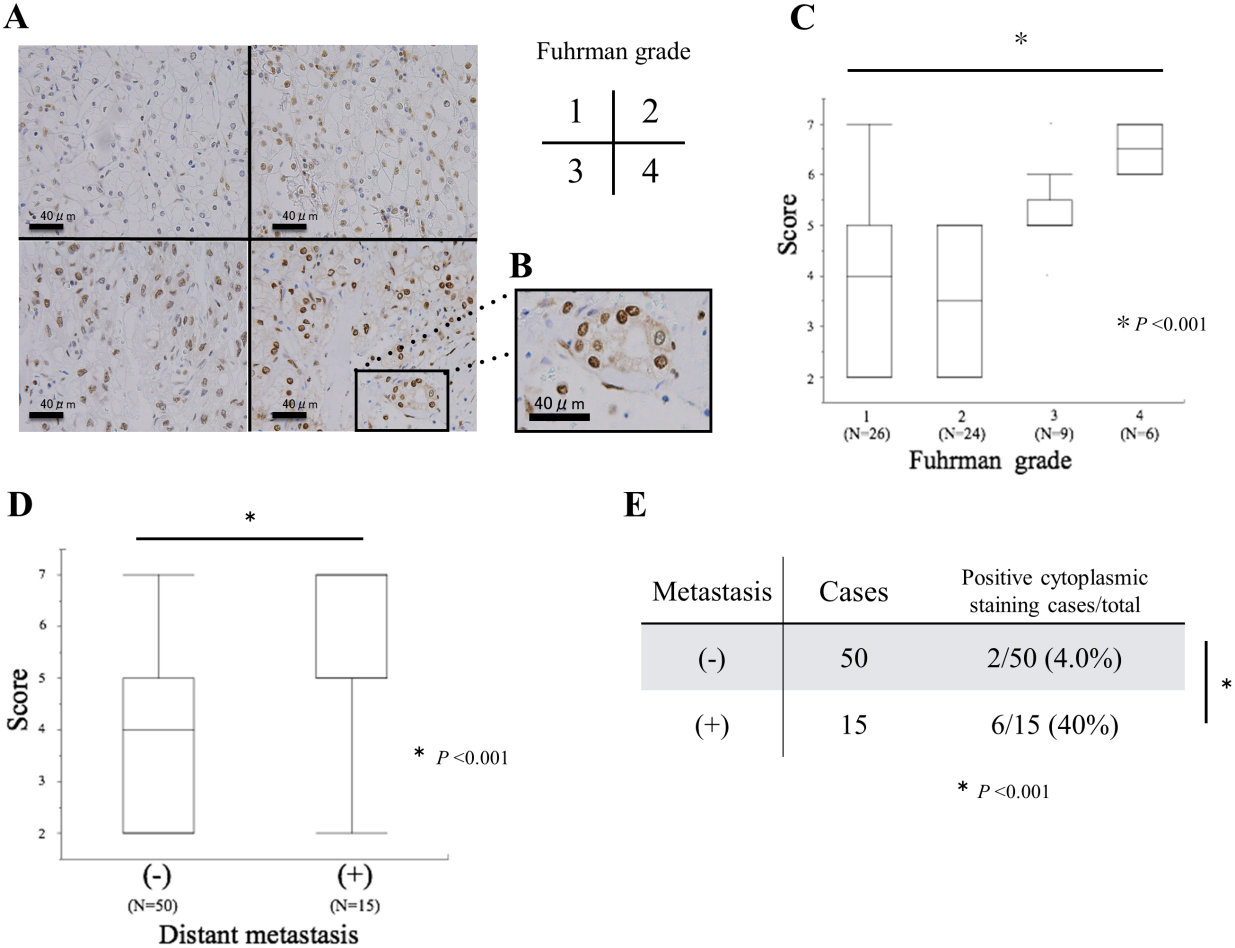
Correlation hnRNP K expression alterations with RCC aggressiveness in IHC analysis. A. Immunoreactivity of hnRNP K in clear cell type (cc) RCC Fuhrman grade 1, 2, 3, and 4; B. A part of the Fuhrman grade 4 micrograph is highly magnified in the right panel; C. Staining scores for hnRNP K in ccRCC tissues with diverse Fuhrman grades; D. Staining scores in primary ccRCC specimens with and without distant metastasis; E. Correlation between cytoplasmic staining and distant metastasis in these ccRCC specimens.

We then performed IHC analysis to evaluate hnRNP K protein expression in paraffin-embedded clear cell type (cc) RCC tissue sections from 65 RCC patients and normal renal specimens from 7 AML patients who underwent nephrectomy, respectively. While normal renal proximal tubule cells showed very weak hnRNP K staining in the nucleus (S2A Fig), the nucleus and/or cytoplasm of ccRCC cells tended to be positive for hnRNP K staining, with a gradual increase according to tumor aggressiveness (Fig 3A). In particular, hnRNP K staining in ccRCC cells with Fuhrman grade 4 was distributed in both the nucleus and cytoplasm (Fig 3A and 3B). Furthermore, we scored both the percentage of positive cells and the intensity of staining from the IHC data in accordance with the protocol, as described in the Materials and Methods section. First, the staining score of normal renal tissue specimens was significantly low compared with that of Fuhrman grade 1 RCC sections (S2B Fig). Next, there was a statistically significant positive correlation between the score of hnRNP K staining and Fuhrman grade (Fig 3C; lane 1–4, Kruskal-Wallis one-way analysis, *P*<0.001). Next, the hnRNP K staining score in primary RCC with distant metastasis was shown to be significantly higher than that in primary RCC without metastasis (Fig 3D; Mann Whitney *U*-test, *P*<0.001). Finally, we analyzed the correlation between cytoplasmic distribution of hnRNP K expression and distant metastasis in these RCC specimens. The rate of positive cytoplasmic hnRNP K staining was significantly higher in primary RCC specimens with metastasis (40%) compared with those without metastasis (4.0%) (Fig 3E; Mann Whitney *U*-test, *P*<0.001).

### TGF-β stimulus increases cytoplasmic distribution of hnRNP K expression in RCC cells

**Fig 4:**
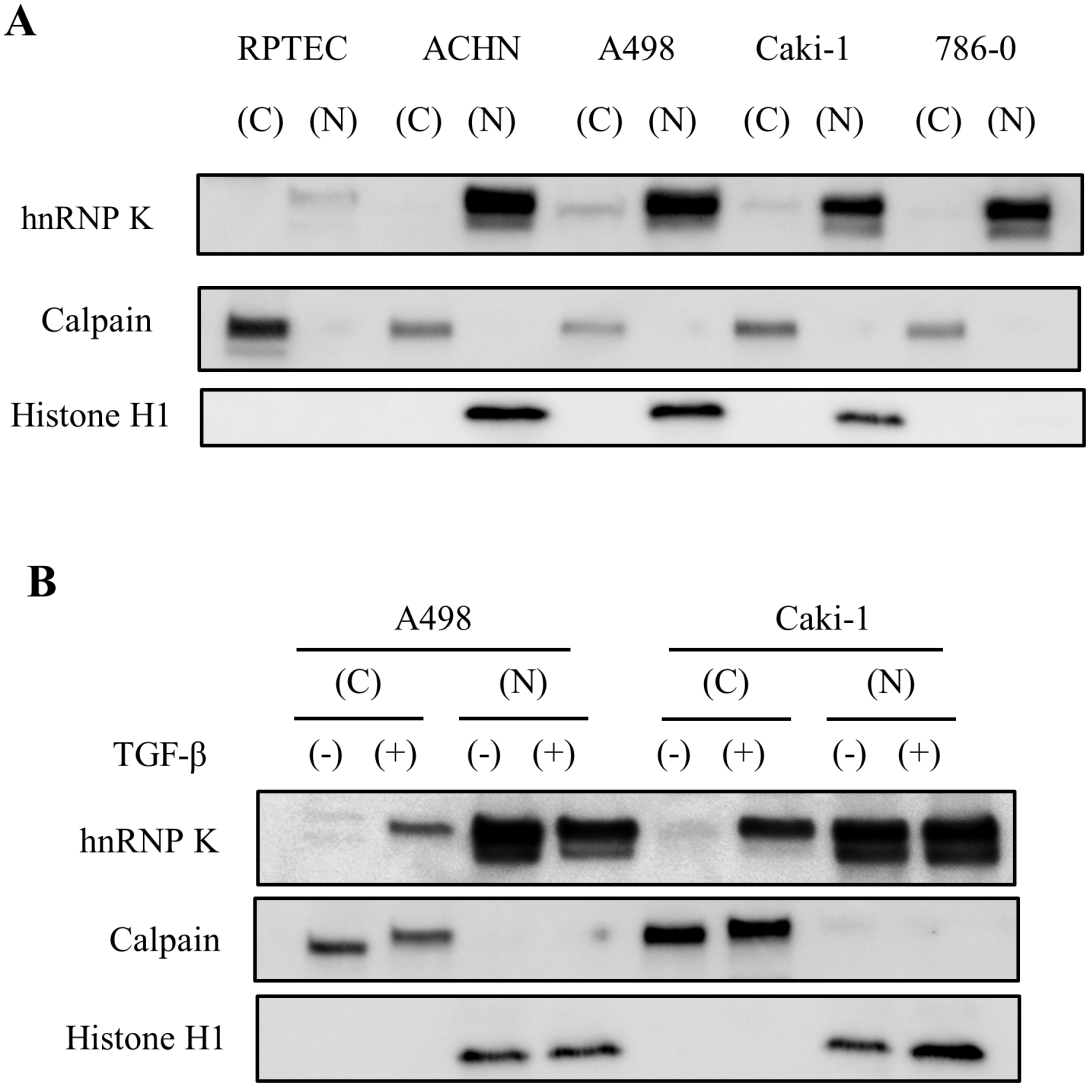
Chagnes in subcelluar distribution of hnRNP K under the condition with TGF-β treatment. A. Evaluation of hnRNP K protein expression in subcellular fractions (C; cytoplasm and N; nucleus) of RPTEC and four RCC cell lines. Expression levels of hnRNP K in cytoplasmic and nuclear fraction are corrected by expression of calpain or histone H1, respectively; B. Estimation of the effect of TGF-β on the subcellular distribution of hnRNP K expression in A498 and Caki-1 cell lines. Each fractioned expression level of hnRNP K is corrected by expression of calpain (cytoplasm) or histone H1 (nucleus).

First, we examined the cellular distribution of hnRNP K protein expression in normal renal cells (RPTEC) and RCC cell lines by Western blot analysis. While there was no detectable hnRNP K expression in the cytoplasmic component of RPTEC (normal renal cells), ACHN and 786–0 RCC cells, both A498 and Caki-1 RCC cells showed weak positive signals in the cytoplasmic fraction compared with the nuclear fraction (Fig 4A). Next, we analyzed the effect of TGF-β on the cellular distribution of hnRNP K protein expression in RCC cells. Stimulation with TGF-β markedly enhanced the cytoplasmic distribution of hnRNP K, without an increase in expression level of hnRNP K in the nuclear component (Fig 4B).

### Down-regulation of hnRNP K expression suppresses TGF-β-mediated cell invasion through inhibition of cytoplasmic distribution of hnRNP K in A498 cells

**Fig 5:**
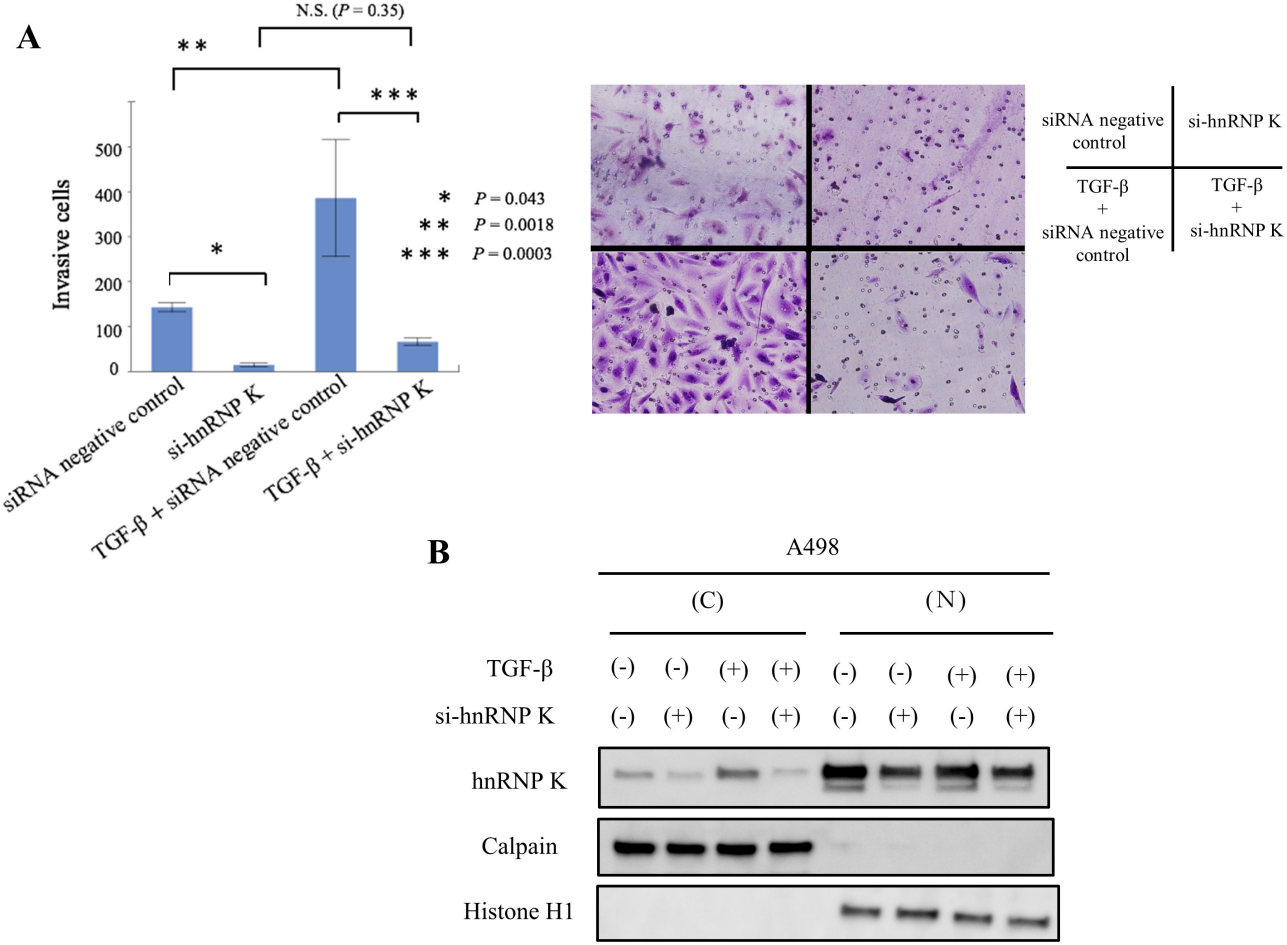
Knock-down of hnRNP K strongly suppresses TGF-β-induced RCC cell invasion. A. Invasion assay in si-hnRNP K-treated A498 cells. Three individual experiments were performed, and mean ± s.d. is presented; *P, **P, ***P<0.05 compared with control (Student’s *t*-test, n = 3). N.S.; no significant difference; B. Effect of hnRNP K knock-down on TGF-β-induced subcellular localization of hnRNP K expression in A498 cells.

We then investigated the effect of hnRNP K knock-down on TGF-β-induced cellular invasiveness in A498 cells at 48 h after transfection with si-hnRNP K. As shown in Fig 1C, there was no significant difference in cell growth between si-hnRNP K-treated cells and control under this condition. Knock-down of hnRNP K significantly decreased cell invasion compared with that in control mock cells (Fig 5A; lane 1 vs. 2 and 3 vs. 4, *P* = 0.043 and *P* = 0.0003, respectively). Treatment with TGF-β caused almost a 2.7-fold increase in the number of invasive mock cells (Fig 5A; lane 1 vs. 3, *P* = 0.0018), and showed no significant difference in the number of invasive hnRNP K-knock-down cells (Fig 5A; lane 2 vs. 4, *p* = 0.35). Next, we examined the amount of hnRNP K knock-down in cellular components (cytoplasm and nucleus) of TGF-β-stimulated A498 cells. Knock-down of endogenous hnRNP K expression markedly inhibited the expression of cytoplasmic hnRNP K under conditions with/without TGF-β treatment (Fig 5B).

### Exogenous expression of mutant hnRNP K with dominant cytoplasmic distribution restores cell invasion inhibited by down-regulation of endogenous hnRNP K expression

**Fig 6:**
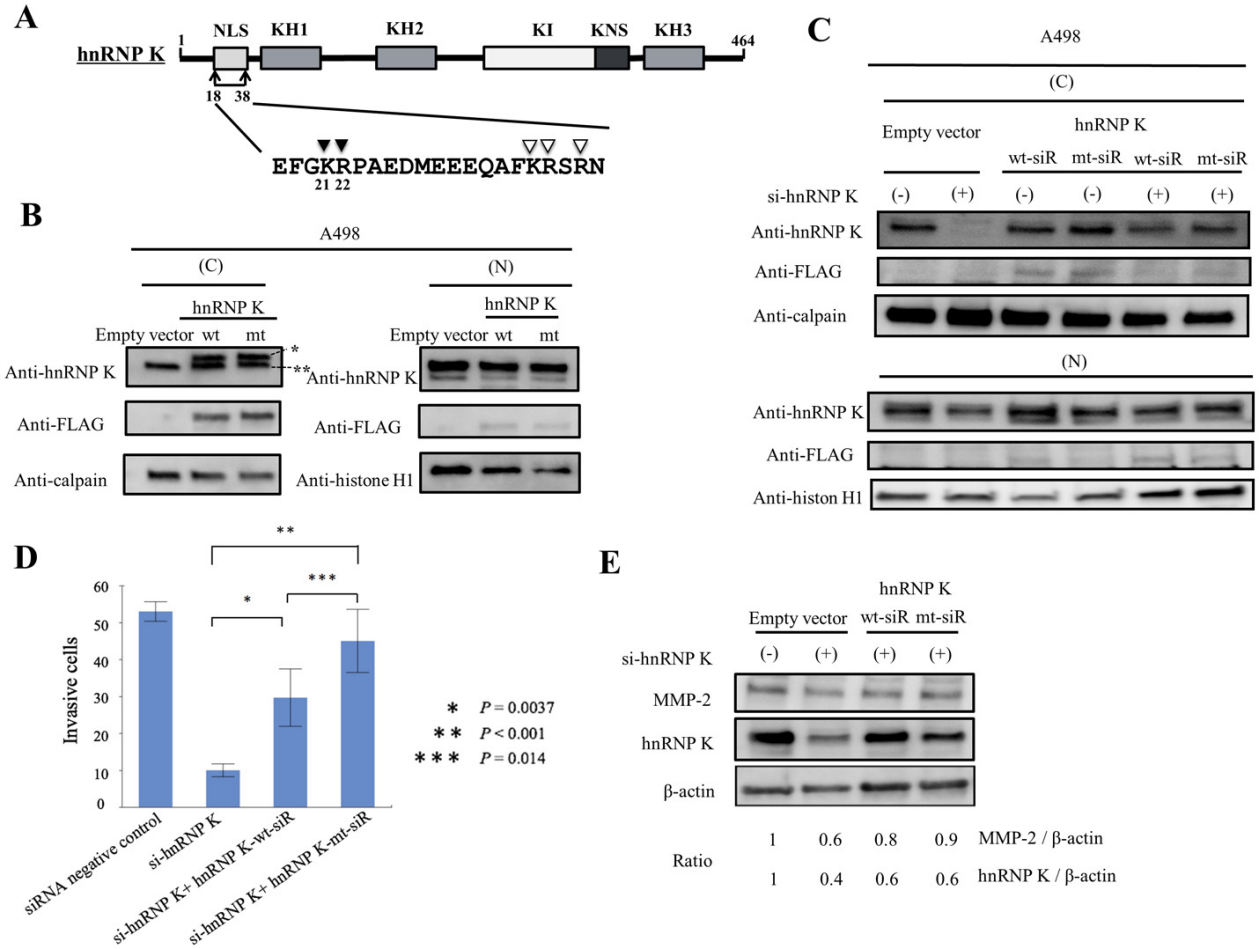
Functional analysis of exogenous hnRNP K mutants in RCC cells with down-regulated endogenous hnRNP K. A. Mapping of structural domains of hnRNP K protein. The locations of the nuclear localization signal (NLS), K homologue (KH), K-protein-interactive (KI) and nuclear shuttling (KNS) domains are indicated (upper panel). A putative consensus sequence of the bipartite NLS is defined as (K/R)(K/R)X10-12(K/R)3/5, where X indicates any amino acid and (K/R)3/5 represents at least three of either lysine or arginine out of five sequential amino acids. *Arrowheads* indicate the conserved amino acid residues in NLS (lower panel); B. Exogenous expression of hnRNP K wild type (wt) or mutant (mt; K21A and R22A) is dominant in the cytoplasm. Single asterisk (*) and double asterisks (**) indicate FLAG-hnRNP K and endogenous hnRNP K, respectively in the left and upper panel. Signals against FLAG-tag are indicated as exogenous expression of hnRNP K; C. Expression of hnRNP K constructs was measured by western blotting with anti-hnRNP K antibody in A498 cells treated with control siRNA or si-hnRNP K. As indicated in the column, these cells were co-transfected with control empty vector, si-hnRNP K resistant wild-type hnRNP K (hnRNP K-wt-siR) or si-hnRNP K resistant mutant hnRNP K (hnRNP K-mt-siR). Anti-calpain antibody or anti-histone H1 antibody was used as loading control for the cytoplasmic or nuclear fraction, respectively; D. Exogenous expression of hnRNP K-wt-siR or hnRNP K-mt-siR significantly suppressed the inhibition of cell invasion by endogenous hnRNP K knock-down in A498 cells. Three individual experiments were performed, and mean ± s.d. is presented; *P, **P, ***P<0.05 compared with the second lane treated with si-hnRNP K (Student’s *t*-test, n = 3); E. Exogenous expression of hnRNP K-wt-siR or hnRNP K-mt-siR reversed the down-regulation of MMP-2 expression induced by endogenous hnRNP K knock-down. Anti-β-actin antibody was used as an indicator of the loaded volume of total cell lysates.

In previous sections, we demonstrated that the cytoplasmic distribution of hnRNP K positively correlated with cancer metastasis or cell invasion of RCC. In this section, we investigated whether exogenous wild-type (wt) FLAG-hnRNP K or mutant (mt) FLAG-hnRNP (K21A and R22A) with regenerated nuclear NLS directly affected the invasiveness of RCC cells. First, these FLAG-tagged (wt and mt) hnRNP K proteins and empty vector (control) were overexpressed in A498 cells. As shown in Fig 6B, both exogenous FLAG-wt-hnRNP K and FLAG-mt-hnRNP K were dominantly expressed in the cytoplasm of A498 cells. Particularly, the expression of FLAG-mt-hnRNP K was more restricted to the cytoplasm than that of FLAG-wt-hnRNP K (Fig 6B; second panel, anti-FLAG antibody). Next, to test the aforementioned hypothesis strictly, we engineered vectors expressing FLAG-hnRNP K (wt or mt) that were resistant to the hnRNP K-targeting siRNA (see Materials and Methodssection). We termed si-hnRNP K resistant FLAG-wt-hnRNP and si-hnRNP K resistant FALG-mt-hnRNP K: hnRNP K-wt-siR and hnRNP K-mt-siR, respectively. These specific vectors expressing hnRNP K-wt-siR or hnRNP K-mt-siR and empty control vector were co-transfected with si-hnRNP K or siRNA control into A498 cells. Furthermore, we analyzed the cellular distribution of exogenous hnRNP K-wt-siR and hnRNP K-mt-siR proteins under the condition with endogenous hnRNP K knock-down in A498 cells. While both hnRNP K-wt-siR and hnRNP K-mt-siR proteins were dominantly expressed in the cytoplasm, the expression of hnRNP K-mt-siR was greatly increased (about 1.7 fold) in the cytoplasm compared with that of hnRNP K-wt-siR (Fig 6C). Both exogenous hnRNP K-wt-siR and hnRNP K-mt-siR proteins could clearly reverse inhibition of cell invasion induced by knock-down of endogenous hnRNPK protein (Fig 6D; lane 2 vs. 3 and lane 2 vs. 4, *P* = 0.0037 and *P*<0.001, respectively). Expression of hnRNP K-mt-siR strongly reversed such suppression of invasion compared with that of hnRNP K-wt-siR (Fig 6D; lane 3 vs 4, *P* = 0.014). Next, we examined the effect of hnRNP K-wt-siR or hnRNP K-mt-siR on regulation of MMP-2, a component of invadopodia in the machinery required for cell invasion, in the aforementioned condition. Exogenous expression of these constructs restored the expression of MMP-2 inhibited by endogenous hnRNP K knock-down, and particularly hnRNP K-mt-siR has more markedly suppressed the inhibition of MMP-2 expression compared with hnRNP K-wt-siR (Fig 6E).

## Discussion

Aberrant upregulation of hnRNP K expression has been reported in various cancers including those of the lung, liver, pancreas, breast, colon and prostate. HnRNP K has been strongly implicated in cancer development and progression via several biological events such as chromatin remodeling, transcriptional regulation, translational control and cellular signal transduction.

However, to our knowledge, there are no reports on the biological functions of hnRNP K underlying tumorigenesis and advancement in RCC. In this study, we initially investigated the expression of hnRNP K protein in several RCC cell lines. Expression levels of hnRNP K were clearly elevated in four RCC cell lines (ACHN, A498, Caki-1 and 786–0) compared with normal renal cells (RPTEC). Knock-down of endogenous hnRNP K protein significantly inhibited cell proliferation of these RCC cell lines compared with control (Fig 1C). Down-regulation of endogenous hnRNP K obviously induced apoptosis at a relatively late time point (72 h after si-hnRNP K transfection) in RCC cells (Fig 2). This phenomenon appears to be similar to the results that have been previously reported in hepatocellular carcinoma cells transfected with si-hnRNP K[37]. Next, we examined the relationship between hnRNP K expression and pathological grade in human RCC specimens. The expression level of hnRNP K in RCC tissues was positively correlated with Fuhrman grade, a potent predictor of tumor progression and recurrence (Fig 3C). Moreover, hnRNP K expression was significantly upregulated in primary RCC samples with distant metastasis, compared with those without metastasis (Fig 3D). In previous reports, the cytoplasmic distribution of hnRNP K protein has been shown to be positively associated with cancer progression and patient prognosis. Also in this IHC analysis, the rate of positive hnRNP K staining in the cytoplasmic component was significantly higher in primary RCC tissues with metastasis compared with those without metastasis (Fig 3E). However, it is limited to some extent to elucidate that tumor metastasis is dominantly related with either an increase of total expression or cytoplasmic accumulation of hnRNP K. Furthermore, we examined the cytoplasmic expression of hnRNP K protein in normal renal cells and four RCC cell lines by Western blot analysis. There were positive signals against hnRNP K protein in the cytoplasm of two RCC cell lines (A498 and Caki-1) (Fig 4A). A previous report demonstrated that the addition of serum after a serum-starved condition for 24 h or exposure to fibronectin induces cytoplasmic accumulation of hnRNP K from its predominant nuclear localization in HT-1080 (fibrosarcoma) cells [38]. In turn, we tested whether treatment with TGF-β, an effector of cell mobility, can promote a change of hnRNP K expression levels in the nucleus and cytoplasm of A498 and Caki-1 cells. Interestingly, this treatment markedly enhanced cytoplasmic accumulation of hnRNP K in these RCC cells, and particularly in A498 cells, there was almost no change in total (nuclear and cytoplasmic) expression level of hnRNP K (Fig 4B). Next, we showed that knock-down of endogenous hnRNP K expression completely suppressed TGF-β-triggered tumor invasion, concomitant with marked inhibition of the cytoplasmic accumulation of hnRNP K expression induced by TGF-β treatment in A498 cells (Fig 5). Finally, we have shown that exogenous FLAG-mt-hnRNP K (K21A and R22A) resistant to si-hnRNP K is dominantly expressed in the cytoplasm and markedly suppresses the inhibition of cell invasion induced by endogenous hnRNP K knockdown (Fig 6C and 6D). MMP-3, MMP-10 and MMP-12 have been shown to be up-regulated in hnRNP-K-overexpressing lung and nasopharyngeal cancer cells [39,40]. Also in RCC cells, we elucidated that MMP-2 expression was down-regulated by endogenous hnRNP K knock-down, and exogenous hnRNP K dominantly expressed in the cytoplasm reversed the inhibition of MMP-2 expression by endogenous hnRNP K knock-down (Fig 6E). In accordance with these experimental results, we found that hnRNP K plays a key role in cell proliferation and apoptosis in RCC cells, and particularly, cytoplasmic expression of hnRNP K is strongly associated with invasive behavior of RCC cells. Furthermore, the detection of cytoplasmic distribution of hnRNP K in IHC analysis appears to be a promising prognostic biomarker to predict metastatic RCC and to identify the high-risk RCC patients requiring systemic therapy.

HnRNP K also has the potential to be a novel target to treat advanced cancer and to prevent distant metastasis in RCC patients. While there are diverse strategies to inhibit hnRNP K-dependent pathways (e.g., p53-mediated transcription, Ras/Raf/MEK/ERK, c-Myc activation), post-translational modification associated with the subcellular localization of hnRNP K is very important for the development of novel therapy targeting hnRNP K. It is expected that in the near future, novel agents based on this concept will be applied as candidates to treat advanced RCC patients.

## Supporting Information

S1 Fig Comparison of the knocking down efficiency among the control siRNA and two independent si-hnRNP K (s6738 and s6739) in A498 cells. (TIF)

S2 Fig A. Immunoreactivity of hnRNP K in normal renal proximal tubules B. Comparison of hnRNP K staining score between normal renal tissue and Fuhrman grade 1 RCC specimens.
(TIF)
