# Supplementary figures and images for: Cytoplasmic Accumulation of Heterogeneous Nuclear Ribonucleoprotein K Strongly Promotes Tumor Invasion in Renal Cell Carcinoma Cells
Source: PLoS One. 2015 Dec 29;10(12):e0145769. doi: 10.1371/journal.pone.0145769 (PMC4699215; doi:10.1371/journal.pone.0145769)

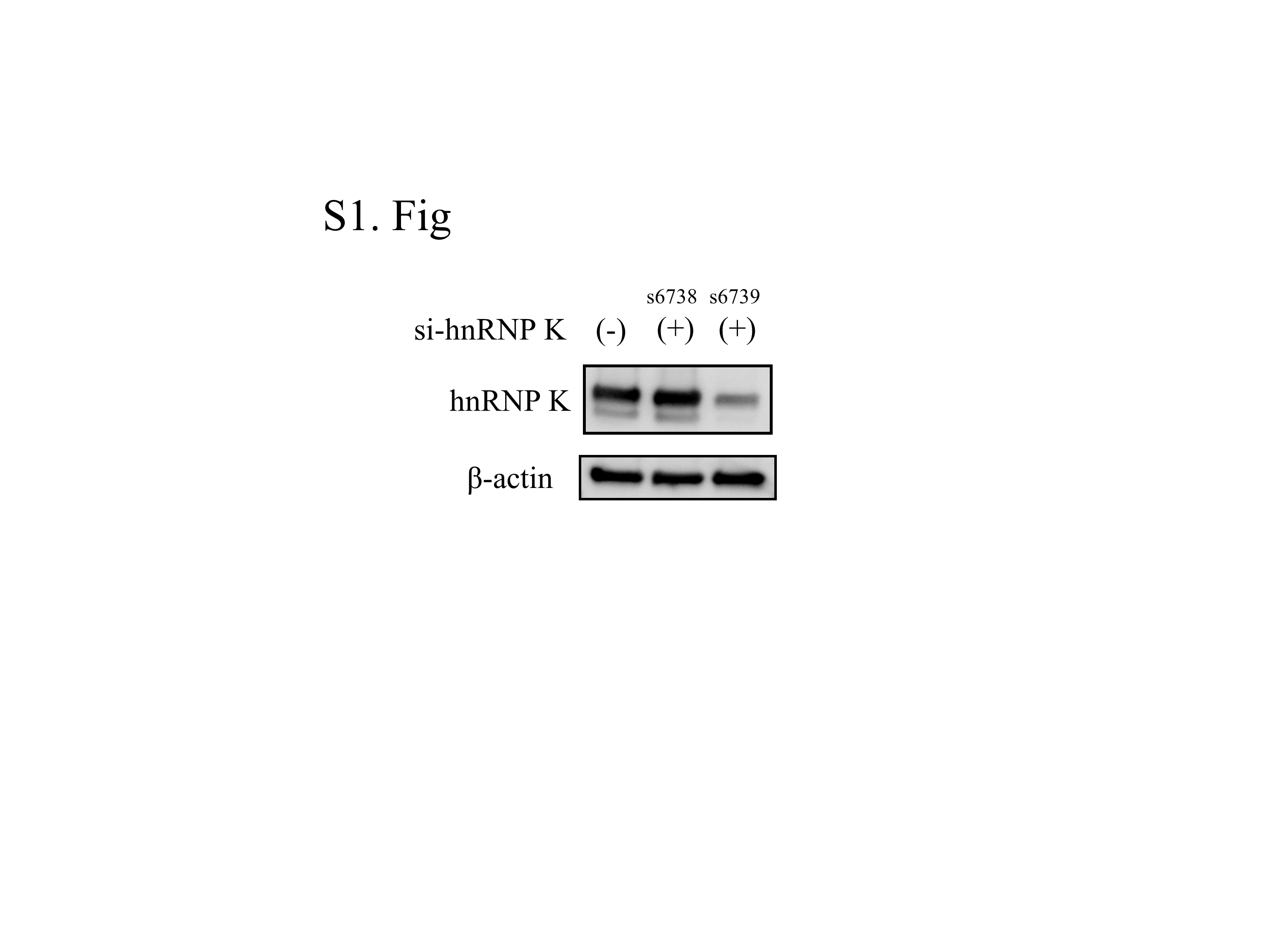

Supplement: S1 Fig — (TIF) [file pone.0145769.s001.tif]

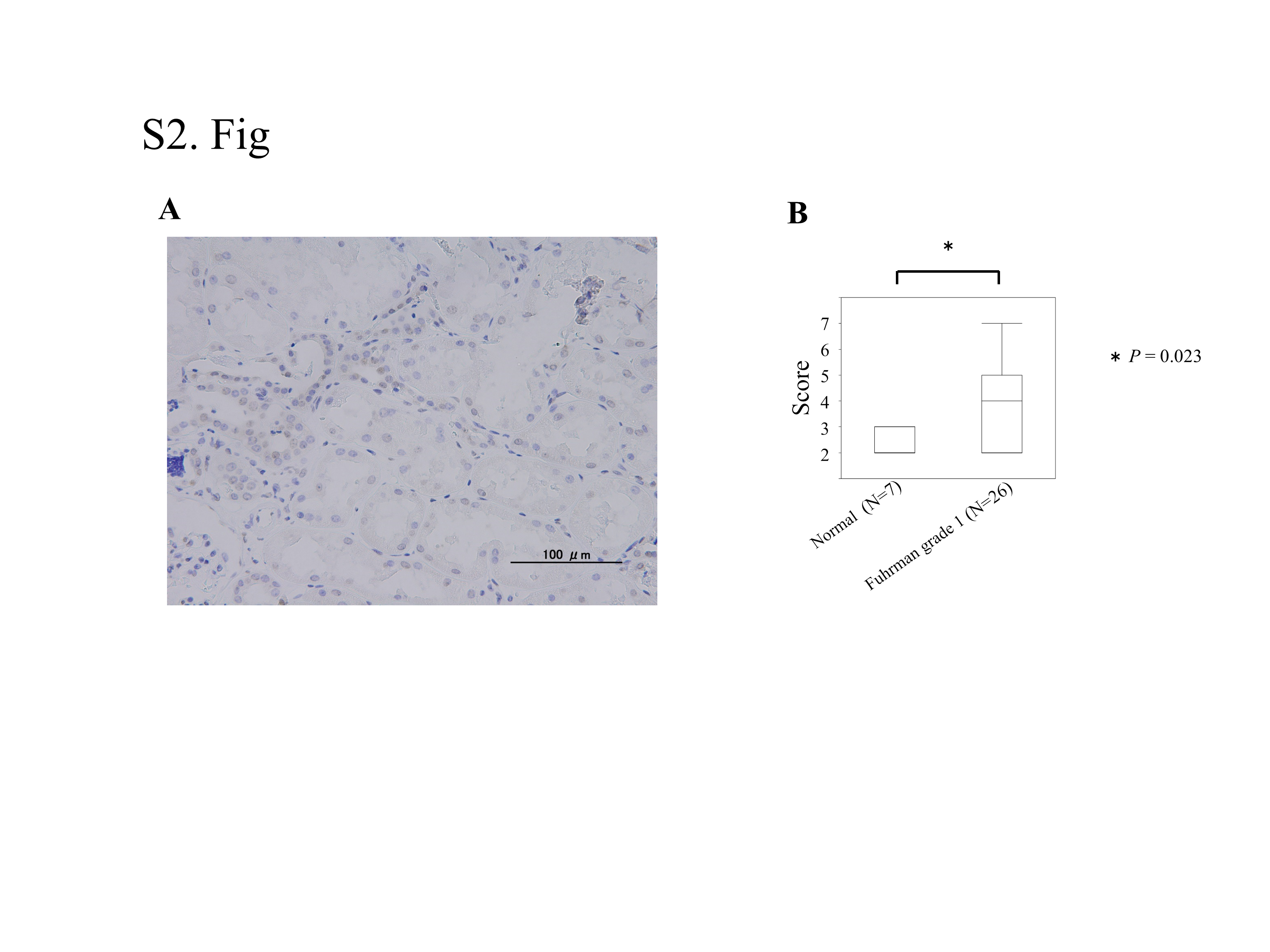

Supplement: S2 Fig — A. Immunoreactivity of hnRNP K in normal renal proximal tubules B. Comparison of hnRNP K staining score between normal renal tissue and Fuhrman grade 1 RCC specimens. (TIF) [file pone.0145769.s002.tif]
